# Visual and ocular findings in children with fetal alcohol spectrum disorders (FASD): validating the FASD Eye Code in a clinical setting

**DOI:** 10.1136/bmjophth-2022-001215

**Published:** 2023-03-02

**Authors:** Lucyn Ayoub, Eva Aring, Emelie Gyllencreutz, Valdemar Landgren, Leif Svensson, Magnus Landgren, Marita Andersson Grönlund

**Affiliations:** 1 Department of Ophthalmology, Faculty of Medicine and Health, Örebro University, Örebro, Sweden; 2 Department of Clinical Neuroscience, Institute of Neuroscience and Physiology, Sahlgrenska Academy at the University of Gothenburg, Gothenburg, Sweden; 3 Region Västra Götaland, Sahlgrenska University Hospital, Department of Ophthalmology, Mölndal, Sweden; 4 Region Västra Götaland, Skaraborg Hospital, Department of Psychiatry, Skövde, Sweden; 5 Gillberg Neuropsychiatry Centre, Institute of Neuroscience and Physiology, University of Gothenburg, Gothenburg, Sweden; 6 Region Västra Götaland, Skaraborg Hospital, Department of Paediatrics, Unit of Neurodevelopmental Disorders, Mariestad, Sweden

**Keywords:** Diagnostic tests/Investigation, Optic Nerve, Optics and Refraction, Vision, Child health (paediatrics)

## Abstract

**Objective:**

Fetal alcohol spectrum disorders (FASD) is an umbrella term covering a spectrum of medical conditions caused by prenatal alcohol exposure. The FASD Eye Code is a new complementary ophthalmological diagnostic tool created to corroborate the complex FASD diagnosis. The aim of this work was to validate the FASD Eye Code by testing it on a second group of children diagnosed with FASD in a clinical setting.

**Methods and analysis:**

A clinical study was carried out in a group of 21 children (13 males, 8 females, mean age 13.3 years) investigated for suspected FASD and a healthy sex-matched and age-matched control group (n=21). The participants underwent a detailed ophthalmological examination including visual perception problems (VPPs) assessment. Clinical examination results were compiled, and total scores were calculated according to the FASD Eye Code protocol (range 4–16).

**Results:**

The median total score in the FASD group was 8. Eight individuals in the FASD group and none of the controls obtained a total score of ≥9 corresponding to 38% sensitivity and 100% specificity with an area under the curve of 0.90. A cut-off total score of ≥8 showed 52% sensitivity and 95% specificity. One individual in the FASD group versus 12 controls had a total score of 4, representing normal findings. No significant difference between the two groups regarding VPPs was seen.

**Conclusion:**

The FASD Eye Code can be used as a complementary diagnostic tool for FASD to assist in diagnosis and to detect ophthalmological abnormalities in individuals with suspected FASD.

**WHAT IS ALREADY KNOWN ON THIS TOPIC**

- Many individuals with fetal alcohol spectrum disorders (FASD) exhibit ophthalmological abnormalities.
- Despite having different sets of diagnostic criteria assessing FASD worldwide, there are currently no complementary ophthalmological diagnostic tools available that independently strengthen an FASD diagnosis.

**WHAT THIS STUDY ADDS**

- Validating the novel complementary ophthalmological diagnostic tool, the FASD Eye Code, on a second group of individuals with suspected FASD shows good clinical reliability, supporting the utility of using this tool in the FASD diagnostic workup.

**HOW THIS STUDY MIGHT AFFECT RESEARCH, PRACTICE OR POLICY**

- Through a structured eye examination using the FASD Eye Code, an FASD diagnosis can be corroborated, and treatable ophthalmological problems can be detected.

## Introduction

Prenatal alcohol exposure (PAE) is associated with several physical and neurobehavioural findings in individuals who have been exposed to alcohol in utero. Research from the 1970s found that children born to mothers who drank alcohol during pregnancy had a pattern of abnormalities.^1 2^ The observed pattern, denoted fetal alcohol syndrome (FAS), included prenatal and postnatal growth restriction, developmental delay and a constellation of craniofacial stigmata.^1–3^ Today, FAS is encompassed under a broad umbrella term—fetal alcohol spectrum disorders (FASD)—alongside other PAE-related diagnoses, such as partial FAS(pFAS), alcohol-related birth defects and alcohol-related neurodevelopmental disorder (ARND).^4–6^

There are a few diagnostic systems for FASD, with somewhat different sets of diagnostic criteria and cut-offs, and therefore, different diagnostic outcomes.^6–12^ This lack of consensus on diagnostic criteria poses a challenge to the FASD diagnosis. Given the importance of an early diagnosis and the difficulty of investigating FASD, having a complementary diagnostic tool could facilitate this diagnostic process.

Children with FASD have been shown to have a high prevalence of ophthalmological abnormalities in previous studies.^12–14^ Subnormal visual acuity (VA), optic nerve hypoplasia (ONH), retinal vascular malformations, visual perception problems (VPPs), refractive errors, strabismus, short palpebral fissures, ptosis, epicanthus and abnormally increased intercanthal distance (ICD) are well documented in FASD.^3–6 12–19^ Thus, an ophthalmological assessment should be an integral part of the FASD diagnostic workup.^12 19^ An independent structured complementary ophthalmological diagnostic tool could strengthen a suspected FASD diagnosis and simultaneously help detect ophthalmological anomalies early.^20^ This could allow the implementation of timely interventions that could prevent or reduce the future impact of such findings on affected individuals.

The FASD Eye Code, proposed in a recent study by Aring *et al*, is a complementary ophthalmological diagnostic tool for FASD.^20^ It consists of four categories, covering four ophthalmological features that are common in FASD: (A) best-corrected VA (BCVA); (B) refraction; (C) strabismus/binocular function and (D) ocular structural abnormalities. Each category is ranked on a scale from 1 to 4, with 1 indicating normal ophthalmological characteristics and 4 representing a strong presence of ophthalmological anomalies.

The FASD Eye Code has been tested on children with FASD, as well as on healthy children, children with attention deficit hyperactivity disorder (ADHD), children born moderate-to-late preterm and children with Silver-Russell syndrome.^20^ An FASD Eye Code cut-off total score of ≥9 showed high specificity (98%) for FASD versus healthy controls, with a sensitivity of 57%.^20^ This tool has only been evaluated on the target population once. Therefore, the aim of this study was to independently validate the FASD Eye Code by testing it on a second group of children and adolescents with FASD in a clinical setting.

## Materials and methods

### Study design and participants

**Figure 1:**
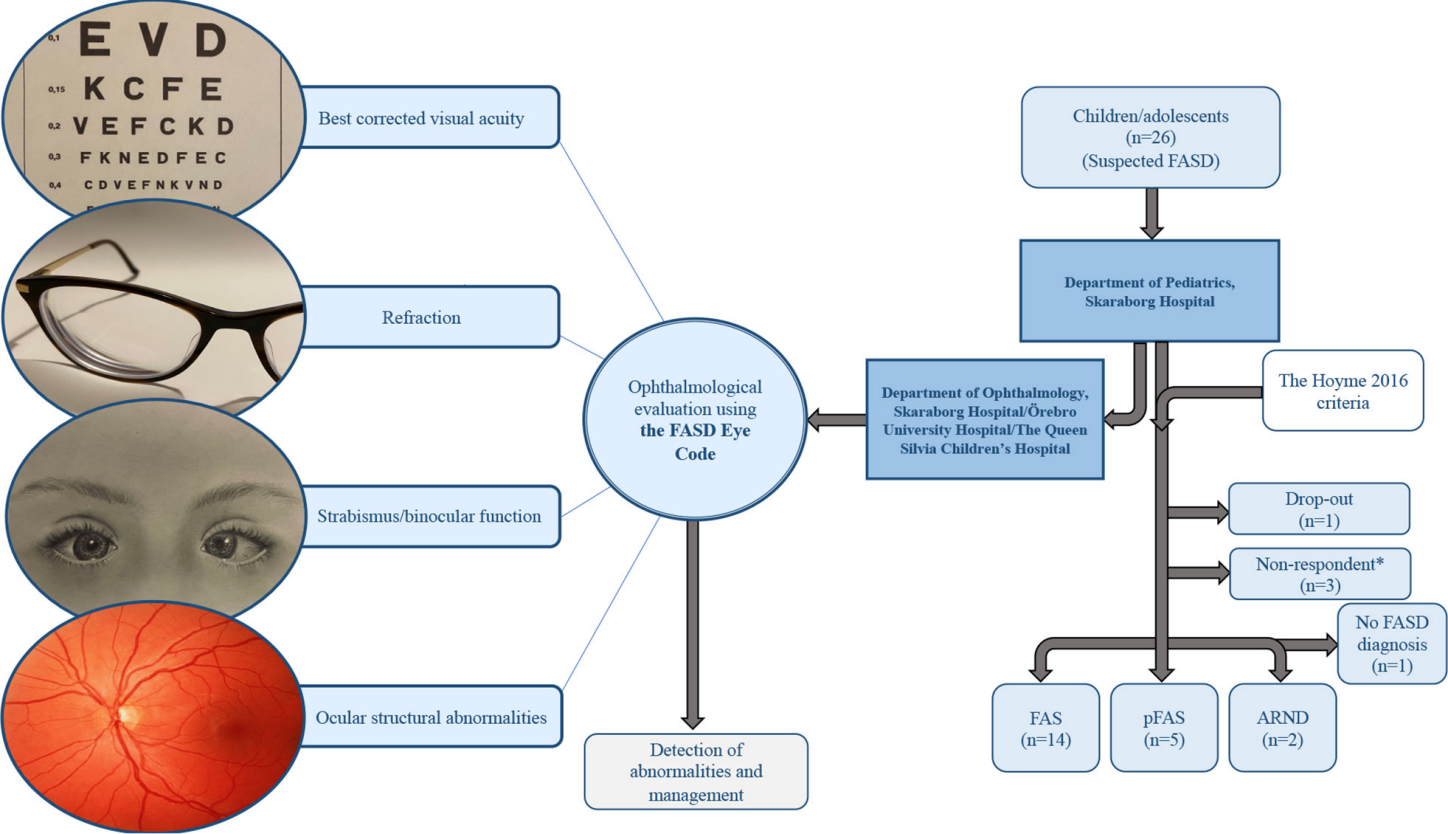
The cohort from which the 21 individuals in the FASD group were drawn and the diagnostic process these individuals went through. FASD, fetal alcohol spectrum disorder. *Individuals who had stated consent verbally, but no written consent could be obtained. ARND, alcohol-related neurodevelopmental disorder; FAS, fetal alcohol syndrome; FASD, fetal alcohol spectrum disorders; pFAS, partial FAS. Illustration: Lucyn Ayoub.

This is a clinical study with both retrospective and prospective aspects. Twenty-six children and adolescents from the Department of Paediatrics (Unit of Neurodevelopmental Disorders), Skaraborg Hospital, Mariestad, Sweden were referred to the Department of Ophthalmology at either Skaraborg Hospital in Skövde, Örebro University Hospital in Örebro or The Queen Silvia Children’s Hospital in Gothenburg, between 2018 and 2021 for further investigation of suspected FASD. These children were later diagnosed with FASD using The Hoyme 2016 criteria,^6^ which were the same criteria used in Aring *et al*’s study.^20^ The same paediatric neurologist and paediatric neuropsychologist examined the whole study population. The diagnostic workup was extensive and the paediatric examination included anthropometric measurements (height, weight and head circumference), dysmorphology, IQ and several behavioural rating scales for attention, activity and adaptation.^6 12 18^ An ophthalmological examination according to the FASD Eye Code was conducted on this group.^20^ In addition, palpebral fissure length (PFL) and ICD were measured, and VPPs were assessed. Twenty-two individuals (13 males, 9 females, mean age 13.4 years, range 9.1–17.8 years) of the 26 initially examined, agreed to participate in this study by providing written consent (figure 1).

Twenty-two healthy schoolchildren (mean age 13.0 years, range 8.6–15.9 years) were chosen from a large reference group to make up an age-matched and sex-matched control group. The reference group consisted of children recruited from day-care centres and schools. These children had undergone the ophthalmological examinations needed for scoring the FASD Eye Code, and their medical background information—including PAE—had been obtained.^21 22^

### Ophthalmological examination

#### VA and refraction

BCVA was tested at 3 m with a linear Konstantin Moutakis VA chart.^23^ The decimal VA was then converted to the logarithm of the minimum angle of resolution (logMAR). BCVA best eye <0.65 decimal (>0.19 logMAR) was considered subnormal.

Refraction was tested under cycloplegia caused by a mixture of phenylephrine (1.5%) and cyclopentolate (0.85%), using an autorefractor (Topcon A6300/KR-8800; Topcon Corporation, Tokyo, Japan). Significant refractive errors were defined as follows: hyperopia ≥2.5 dioptres (D), spherical equivalents (SE), myopia ≥0.5 D SE, anisometropia ≥1.0 D SE and astigmatism ≥1.0 D.

#### Eye motility and stereoacuity

Cover test was used to diagnose strabismus.^20 22^ Binocular function was measured with the TNO random dot stereotest or Lang stereotest.^22 24^ The latter test was used on children unable to participate in the TNO stereotest.

#### Ocular structural abnormalities

A slit-lamp examination and indirect ophthalmoscopy were performed. Left and right PFL and ICD were measured with a ruler.

#### Visual perception

VPPs were assessed using a structured clinical history-taking model,^16 25^ with 12 selected questions focusing on VPPs evaluation in five different areas: recognition, orientation, depth perception, movement perception and simultaneous perception.

### The FASD Eye Code

The results of the clinical examination were compiled, and the total scores and item scores were calculated according to the FASD Eye Code protocol.^20^ Individuals with multiple abnormalities within the same category of the FASD Eye Code obtained the score corresponding to the abnormality with the highest rank according to the FASD Eye Code.^20^

### Statistical analysis

All statistical analyses were performed using SPSS Statistics V.28.0. Descriptive statistics such as means, SD, medians, ranges, percentages, p values and 95% CIs were calculated and used to present background information and results, as appropriate. Cases and controls were matched using the Pocock and Simon minimisation method.^26^ Fisher’s exact test was used to compare the two groups when the outcome variables were dichotomous. The Mann-Whitney U test was used for continuous outcome variables that were not normally distributed. The FASD Eye Code’s ability to discriminate between individuals with FASD/FAS and healthy controls was evaluated, and diagnostic indices such as, specificity, sensitivity, accuracy and likelihood ratio for each cut-off score were reported in classification tables and depicted in receiver operating characteristic (ROC) curves using R V.3.6.3. The pROC package was used to build the ROC curves.^27^ Bonferroni adjustment for multiple testing was preformed, setting the p<0.0045 instead of <0.05.

### Patient and public involvement

Patients and the public were not involved in this research’s design, conduct, reporting or dissemination plans.

## Results

Of the 22 individuals in the suspected FASD group evaluated in this study, 21 individuals received an FASD diagnosis after clinical assessment using the Hoyme 2016 criteria (figure 1).^6^ One individual did not meet the Hoyme 2016 criteria^6^ and did not receive an FASD diagnosis. This individual and the matched control were therefore excluded from further evaluation.

Consequently, the final evaluated study population consisted of 42 children and adolescents: 21 individuals diagnosed with FASD (13 males, 8 females, mean age 13.3 years (range 9.1–17.8 years)) and their 21 healthy age-matched and sex-matched controls (mean age 13.0 years (range 8.6–15.9 years)). The obtained FASD diagnoses were FAS (n=14), pFAS (n=5) and ARND (n=2) (figure 1).

### Ophthalmological findings

#### BCVA, refraction, strabismus/binocular function and ocular structural abnormalities

None of the study participants had a BCVA best eye <0.65 decimal (>0.19 logMAR). Refractive errors were found in 13 individuals diagnosed with FASD and in 6 controls (p=0.06). The refractive data of the study participants are presented in online supplemental file 1. Moreover, when comparing individuals with FASD with controls, heterotropia was more commonly found among those with FASD. However, after Bonferroni correction, this was found to be insignificant (table 1). Subnormal stereoacuity was more common in individuals with FASD, even after Bonferroni correction (p<0.001). However, when the two groups were compared in terms of stereoacuity without the presence of heterotropia, this difference was insignificant after Bonferroni adjustment (p=0.021). Furthermore, a difference in the prevalence of ocular structural abnormalities was observed when comparing individuals with FASD with controls (p<0.001); ocular structural abnormalities were only found in individuals with FASD, with retinal vascular tortuosity being the most common. No significant difference between the two groups regarding ICD or PFL was found (table 1).

10.1136/bmjophth-2022-001215.supp1 Supplementary data

**Table 1:** Ophthalmological findings in 21 individuals diagnosed with FASD and 21 healthy age-matched and sex-matched controls

Characteristics	FASD (n=21)*	Controls (n=21)*	P value
BCVA logMAR RE—mean±SD	0.10±0.18	−0.71±0.56	<0.001
BCVA logMAR LE—mean±SD	0.11±0.20	−0.50±0.069	<0.001
Refraction SE D RE—median (range)	+0.75 (−4.25 to+9.50)	+0.63 (−0.63 to+5.50)	0.95
Refraction SE D LE—median (range)	+0.75 (−4.38 to+9.38)	+0.63 (−0.50 to+6.50)	0.86
Refractive errors—n (%)	13 (62)	6 (29)	0.06
Hyperopia (≥2.5 D SE)—n (%)	7 (33)	3 (14)	0.28
Myopia (≥0.50 D SE)—n (%)	5 (24)	1 (5)	0.18
Anisometropia (≥1.0 D SE)—n (%)	3 (14)	2 (10)	1.000
Astigmatism (≥1.0 D)—n (%)	6 (29)	2 (10)	0.24
Strabismus—n (%)	15 (71)	8 (38)	0.06
Heterophoria—n (%)	8 (38)	7 (33)	1.00
Heterotropia—n (%)	7 (33)	1 (5)	0.045
Subnormal stereoacuity†—n (%)	12 (60) (n=20)	1 (5)	<0.001
With heterotropia—n (%)	7 (35)	1 (5)	0.021
Without heterotropia—n (%)	5 (25)	0	0.021
Structural abnormalities—n (%)	12 (57)	0	<0.001
Ptosis—n (%)	1 (7) (n=15)	0	0.42
Epicanthus—n (%)	2 (15) (n=13)	0	0.14
Retinal vascular abnormalities—n (%)	8 (38)	0	0.003
Optic disc abnormalities—n (%)	3 (14)	0	0.23
ONH—n (%)	2 (10)	0	0.49
PFL mm RE—mean±SD	26.67±1.92 (n=15)	26.3±1.72 (n=20)	0.56
PFL mm LE—mean±SD	26.53±1.89 (n=15)	26.4±1.70 (n=20)	0.93
ICD mm—mean±SD	29.93±2.37(n=14)	29±2.05 (n=20)	0.36

Fisher’s exact test and Mann-Whitney U test were used to compare the two groups’ results.

Bold values are statistically significant.

*When the number of individuals differs from the total number of participants in the group, this is given separately for each category.

†Stereoacuity >60” .

BCVA, best-corrected visual acuity; D, dioptre; FASD, fetal alcohol spectrum disorders; ICD, intercanthal distance; LE, left eye; LogMAR, logarithm of the minimum angle of resolution; ONH, optic nerve hypoplasia; PFL, palpebral fissure length; RE, right eye; SE, spherical equivalent.

#### Visual perceptual problems

Fourteen individuals of the 21 diagnosed with FASD had been evaluated for VPPs; 4 participants reported VPPs in 1 or more areas: orientation (n=2), depth perception (n=1) and both orientation and recognition (n=1). Twenty out of the 21 controls had been evaluated for VPPs; 1 participant in that group reported VPPs (orientation). This difference was, however, not statistically significant (p=0.13).

### FASD Eye Code scores

**Figure 2:**
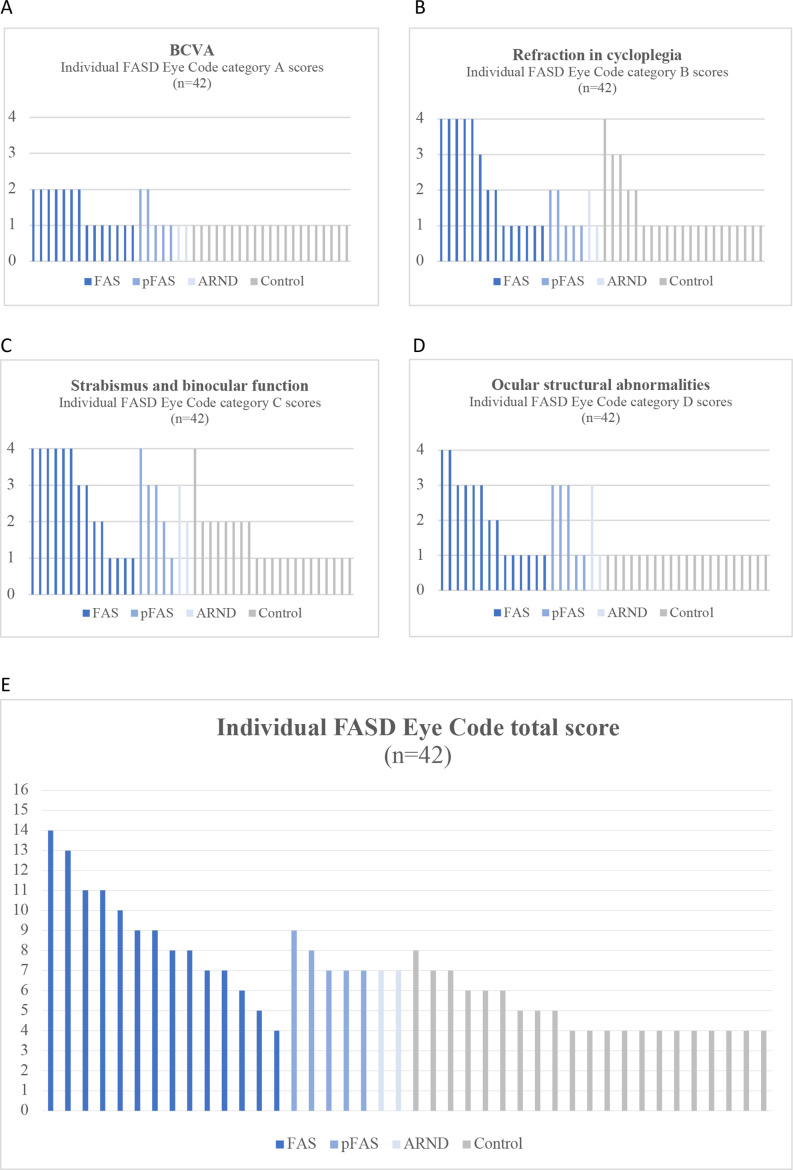
(A–E) Individual FASD Eye Code item scores and total scores in the study population. FASD, fetal alcohol spectrum disorder. ARND, alcohol-related neurodevelopmental disorder; BCVA, best corrected visual acuity; FAS, fetal alcohol syndrome; pFAS, partial FAS.

The individual FASD Eye Code scores are presented in figure 2A–E. The median FASD Eye Code total score in the FASD group was 8 (range 4–14) and that of the control group was 4 (range 4–8). Eight participants in the FASD group obtained a total score of ≥9. Of these eight individuals, seven were diagnosed with FAS and one with pFAS. No one in the control group had a total score greater than 8. Normal ophthalmological findings with a total score of 4 were found in one individual in the FASD group vs 12 controls (figure 2E). The individual in the FASD group that did not receive an FASD diagnosis and was excluded had a total score of 8.

Reduced BCVA, according to the FASD Eye Code’s definition, was found in a total of nine individuals in the FASD group (FAS n=7, pFAS n=2) (figure 2A). As presented in figure 2 B, 11 participants with FASD vs 5 controls had refractive errors corresponding to an item score of ≥2 in category B.

### ROC curve analysis

**Figure 3:**
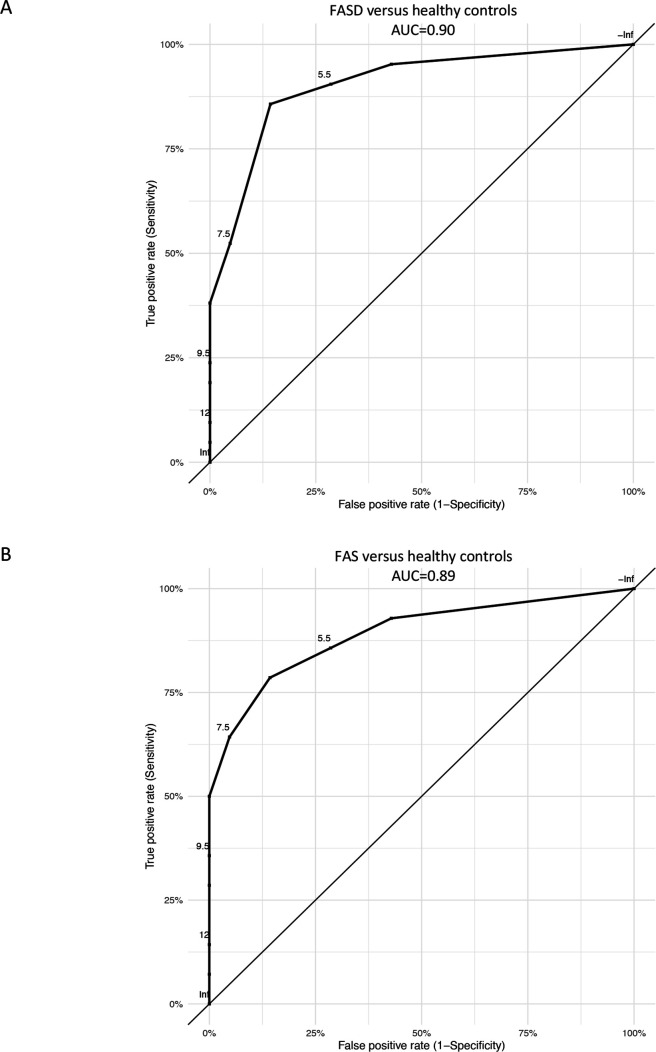
(A–B) Receiver operating characteristic (ROC) curve analyses showing the FASD Eye Code’s ability to differentiate between (A) individuals diagnosed with FASD and healthy sex-matched and age-matched controls and (B) individuals diagnosed with FAS and healthy sex-matched and age-matched controls. FAS, fetal alcohol spectrum; FASD, fetal alcohol spectrum disorder.

The FASD Eye Code had an area under the curve (AUC) of 0.90 (95% CI 0.81 to 1.00) in separating individuals with FASD from controls. A cut-off total score of ≥9 displayed 38% sensitivity and 100% specificity in differentiating individuals with FASD from healthy controls. A cut-off total score of ≥8, on the other hand, showed 52% sensitivity and 95% specificity; for a cut-off total score of ≥7, both the sensitivity and specificity were 86% (figure 3A, online supplemental file 2). For FAS versus healthy controls, the FASD Eye Code had an AUC of 0.89 (95% CI 0.74 to 1.00) (figure 3B, online supplemental file 2).

10.1136/bmjophth-2022-001215.supp2 Supplementary data

## Discussion

Diagnostic assessment of FASD is challenging and relies on a multidisciplinary evaluation approach due to the heterogenic nature of FASD. Additionally, individuals with FASD have a high prevalence of ophthalmological abnormalities.^12–14 19^ Implementing a complementary ophthalmological diagnostic tool in the diagnostic workup of FASD is warranted.

The results of this study showed significant differences in BCVA, stereoacuity and structural abnormalities when comparing the two groups. These findings indicate that these categories chosen to build the FASD Eye Code are reasonable even in the age group validated in this study. Regarding refraction, the difference in refractive errors between the two groups was not significant. This indicates that category B in the FASD Eye Code might need some adjustment regarding age and cut-off values for refractive errors.

Our results showed that the specificity of the FASD Eye Code was higher than its sensitivity for the cut-off total score of ≥9, which is consistent with the results of Aring *et al*’s study.^20^ This indicates that individuals obtaining higher total scores have an increased likelihood of having PAE as the prime aetiological factor behind their ophthalmological abnormalities, although other possible causes should still be investigated in parallel.^20^ On the other hand, an FASD diagnosis cannot be ruled out when an individual scores lower than 9, which is supported by the fact that about two-thirds of our FASD group scored lower than 9. A cut-off total score of ≥8 resulted in the capture of three more individuals with FASD and one control. In comparison, a cut-off total score of ≥7 instead of ≥9 captured 10 more individuals with FASD and 3 controls. However, the FASD Eye Code should only be used on children being evaluated for suspected FASD, because it is proposed as a complementary diagnostic tool and not as a screening tool, and the overall clinical picture must be taken into consideration. Our ROC curve analysis suggested that a cut-off total score of ≥8 seems to provide reasonable specificity and sensitivity and would likely help in detecting individuals with less severe forms of FASD.

Our results displayed a wide variation in total scores among the study participants, particularly in the FASD group, with the obtained total scores in this group ranging from 4 to 14. This indicates that the severity of ophthalmological involvement in FASD may vary drastically. Regardless of the primary cause behind these findings and whether these individuals reach the cut-off total score or not, abnormalities detected with the FASD Eye Code are important and need clinical management. This study further supports the utility of implementing a complementary ophthalmological diagnostic tool in the FASD diagnostic workup.

The individual in the suspected FASD group who did not receive an FASD diagnosis and was thus excluded from further evaluation had ophthalmological findings that were severe enough to reach a total score of 8. The individual in question had ADHD, which can be associated with ophthalmological abnormalities.^28 29^ An ROC curve analysis by Aring *et al* showed an AUC of 0.66 for FASD versus ADHD, which was smaller than the AUC for FASD versus controls,^20^ and this could explain this case. Furthermore, there is a possibility that this individual would meet the criteria for an FASD diagnosis if evaluated using a different diagnostic system.^6 7 10 11^

Strabismus, retinal vascular abnormalities and ONH in children with FASD are known to persist into adulthood.^15^ The mean BCVA shown in our FASD group was similar to the mean BCVA observed in a group of young adults with FASD in a study by Gyllencreutz *et al*.^15^ The FASD group evaluated in that study had even been evaluated in childhood (mean age 7.8 years), and a higher mean logMAR BCVA (lower decimal BCVA) than that of our FASD group was observed. Furthermore, our FASD group was more nearsighted than Gyllencreutz *et al*’s FASD group in childhood but more farsighted than Gyllencreutz’s FASD group in adulthood. This comparison shows that VA and refraction are age-dependent even in individuals with FASD.^21 30 31^ Aring *et al*’s FASD group was evaluated using the FASD Eye Code in childhood (aged 4.9–10.5 years) and in young adulthood (aged 19–28 years); these individuals’ total scores were stable into adulthood, and the FASD Eye Code still showed comparable performance despite the age differences.^20^ Similar performance was displayed in our FASD group, which consisted of older children (aged 9.1–17.8 years).

Some of the typical craniofacial abnormalities associated with FASD that are clinical criteria for FASD have been shown to diminish over time.^18 32^ In this study, no significant differences in ICD or PFL measures between the two groups were found. This emphasises the need of independent complementary diagnostic tools for FASD.

A previous study by Gyllencreutz *et al* showed statistically significant thinning of the retinal nerve fibre layer (RNFL) in adults with FASD, visualised on optical coherence tomography (OCT).^33^ Based on our own clinical experience, some individuals might show significant RNFL thinning on OCT despite not showing any structural abnormalities when examined with a slit-lamp or indirect ophthalmoscopy. Including OCT in the FASD Eye Code could help capture RNFL thinning, and thus increase the sensitivity of this tool. This addition could work in a clinic, but it might be more difficult to apply in studies conducted outside the clinic and in resource-challenged environments, where the size of these devices or their costly nature usually poses a challenge. Recent technical advancements have resulted in handheld OCT devices with comparable performance,^34^ which might facilitate the future adoption of OCT into the FASD Eye Code.

A previous study by Gyllencreutz *et al* showed that VPPs are more common in young adults with FASD.^16^ Increased VPP prevalence—although not statistically significant—was also observed in the FASD group in this study when comparing individuals diagnosed with FASD with controls. Despite missing results from seven individuals with FASD versus one control, four cases of VPPs in children with FASD versus one case in the control group were detected. The difference not being statistically significant could be due to the small sample size analysed or to the fact that one-third of the FASD group had not been evaluated for VPPs. VPPs evaluation could be a sensible addition to the FASD Eye Code. However, this would require having the questions translated and validated in different languages to ensure adequate answers.

The main strengths of our study are that it was conducted using the same methods and diagnostic system used in Aring *et al*’s study and that our study population was examined by the same multidisciplinary team involved in Aring’s research.^20^ This team is familiar with FASD and has conducted many studies on this topic.^12 14–18 20 33^ Furthermore, some potential confounders such as age and sex were taken into consideration in the study design and were minimised by matching individuals with FASD to healthy controls of the same age and sex.

Nevertheless, our study has limitations. The study population is small, which might increase the risk for random errors. All individuals in this study, including the controls, were thoroughly investigated regarding PAE and perinatal and neuropediatric history.^21 22^ However, our control group could have individuals with undetected PAE. Moreover, our FASD group might not be an accurate representation of the general FASD population: the individuals in our FASD group might have been initially recognised and referred for further investigation due to having more noticeable or severe FASD symptoms.

We have made some clarifications and improvements to the FASD Eye Code protocol in order to avoid misinterpretations.^20^ In online supplemental file 3, we present the FASD Eye Code 2.0. The results obtained in Aring *et al*’s study and in this study using the original FASD Eye Code would have been the same regardless of the version of the FASD Eye Code used in the clinical assessment.

10.1136/bmjophth-2022-001215.supp3 Supplementary data

In conclusion, our findings emphasise the importance of an ophthalmological evaluation in suspected FASD. The FASD Eye Code showed good diagnostic performance in this study, with a high AUC, and our results suggest that lowering the cut-off total score from 9 to 8 could help in detecting individuals with milder forms of FASD. However, the FASD Eye Code requires further testing on larger FASD groups, preferably of different ethnic backgrounds and corresponding controls, as well as on additional groups with clinical conditions not attributed to PAE.

## Data Availability

Data are available on reasonable request. All data relevant to the study are included in the article or uploaded as online supplemental information.
