# Supplementary material for: Visual and ocular findings in children with fetal alcohol spectrum disorders (FASD): validating the FASD Eye Code in a clinical setting
Source: BMJ Open Ophthalmol. 2023 Mar 2;8(1):e001215. doi: 10.1136/bmjophth-2022-001215 (PMC9990666; doi:10.1136/bmjophth-2022-001215)
Supplement: Supplementary data [file bmjophth-2022-001215supp001.pdf]

Supplemental file 1: The refractive data of the study participants presented as: sphere/cylinder x axis

FASD		
**Case number**	**Right eye**	**Left eye**
**Case 1**	+0.50/-0.25x151	+0.75/-0.25x2
**Case 2**	+3.75/0x0	+4.25/-0.50x20
**Case 3**	+2.75/0x0	+2.75/0x0
**Case 4**	+0.75/0x0	+0.75/0x0
**Case 5**	+6.25/- 2x170	+5.25/-2x180
**Case 6**	+0.75/-0.75x75	+0.25/-0.50x100
**Case 7**	+1.25/-0.25x8	+1.75/-1x2
**Case 8**	+11/-3x3	+10.75/-2.75x179
**Case 9**	+0.75/-0.50x164	+0.75/-0.25x163
**Case 10**	+2.25/-0.50x37	+1.50/-0.25x148
**Case 11**	+0.25/-0.25x91	+0.25/0x0
**Case 12**	+1/0x0	+1/0x0
**Case 13**	-0.75/-0.25x21	0/-0.50x21
**Case 14**	-0.50/-0.50x171	-0.75/0x0
**Case 15**	+4.50/-2x170	+3/-1x10
**Case 16**	+0.50/-0.75x80	-0.25/-0.25x100
**Case 17**	+3.75/-0.25x69	+2.50/-0.25x0
**Case 18**	-0.25/-1.75x4	0/-1.25x169
**Case 19**	+2.75/-1.50x10	+3/-1x160
**Case 20**	-1.50/-0.50x0	-2/-0.25x0
**Case 21**	-4.25/0x0	-4/-0.75x187

Controls		
**Control number**	**Right eye**	**Left eye**
**Control 1**	+0.50/-1.25x87	+0.75/-0.25x135
**Control 2**	+1.75/-1x80	+1.25/-1x87
**Control 3**	+1.50/-0.25x56	+1.75/-0.25x147
**Control 4**	+1.50/-0.50x101	+1.25/-0.25x80
**Control 5**	+0.75/-0.25x18	+0.75/-0.50x174
**Control 6**	+0.50/-0.25x135	+0.50/-0.25x135
**Control 7**	+0.50/-0.25x95	+0.75/-0.25x122
**Control 8**	+1.75/-0.50x93	+2/-0.50x71
**Control 9**	0/-0.75x85	0/-0.50x89
**Control 10**	-0.25/-0.75x98	-0.25/-0.50x93
**Control 11**	+0.75/-0.50x77	+0.75/-0.50x69
**Control 12**	0/-0.25x77	+0.25/0x0
**Control 13**	+3.25/-0.25x26	+4.25/-0.25x91
**Control 14**	+0.50/-0.25x56	+0.75/-0.25x178
**Control 15**	+2.75/-0.50x73	+3/-0.25x102
**Control 16**	+5.50/0x0	+6.50/0x0
**Control 17**	+2.25/-0.25x99	+2.25/-0.25x84
**Control 18**	+1/-0.25x87	+1/-0.25x120
**Control 19**	+0.50/-0.25x109	+0.50/-0.25x64
**Control 20**	+0.75/-0.25x79	+0.75/-0.50x84
**Control 21**	+0.50/-0.25x122	+0.75/-0.25x43
