# Supplementary material for: Visual and ocular findings in children with fetal alcohol spectrum disorders (FASD): validating the FASD Eye Code in a clinical setting
Source: BMJ Open Ophthalmol. 2023 Mar 2;8(1):e001215. doi: 10.1136/bmjophth-2022-001215 (PMC9990666; doi:10.1136/bmjophth-2022-001215)
Supplement: Supplementary data [file bmjophth-2022-001215supp002.pdf]

Supplemental file 2: The classification tables corresponding to the receiver operating characteristic curve analyses

**FASD versus healthy controls**					
Threshold	Specificity	Sensitivity	Accuracy	Positive likelihood ratio	Negative likelihood ratio
**-∞**	0%	100%	50%	1.0	
**4.5**	57%	95%	76%	2.2	0.1
**5.5**	71%	90%	81%	3.2	0.1
**6.5**	85%	86%	86%	6.0	0.2
**7.5**	95%	52%	74%	11.0	0.5
**8.5**	100%	38%	69%	∞	0.6
**9.5**	100%	24%	62%	∞	0.8
**10.5**	100%	19%	60%	∞	0.8
**12**	100%	10%	55%	∞	0.9
**13.5**	100%	5%	52%	∞	1.0
∞	100%	0%	50%		1.0

**FAS versus healthy controls**					
Threshold	Specificity	Sensitivity	Accuracy	Positive likelihood ratio	Negative likelihood ratio
**-∞**	0%	100%	40%	1	
**4.5**	57%	93%	71%	2.17	0.1
**5.5**	71%	86%	77%	3	0.2
**6.5**	85%	79%	83%	5.5	0.3
**7.5**	95%	64%	83%	13.5	0.4
**8.5**	100%	50%	80%	∞	0.5
**9.5**	100%	36%	74%	∞	0.6
**10.5**	100%	29%	71%	∞	0.7
**12**	100%	14%	66%	∞	0.9
**13.5**	100%	7%	63%	∞	0.9
∞	100%	0%	60%		1.0
