# Supplementary material for: Visual and ocular findings in children with fetal alcohol spectrum disorders (FASD): validating the FASD Eye Code in a clinical setting
Source: BMJ Open Ophthalmol. 2023 Mar 2;8(1):e001215. doi: 10.1136/bmjophth-2022-001215 (PMC9990666; doi:10.1136/bmjophth-2022-001215)
Supplement: Supplementary data [file bmjophth-2022-001215supp003.pdf]

Supplemental file 3: The FASD Eye Code 2.0

	**FASD Eye Code Protocol**	
**Patient name**		**Patient maximum score**
**Code score**	**A. Best corrected visual acuity (BCVA)**	**…/4**
1	BCVA worst eye logMAR ≤0.1 (decimal ≥0.8)	
2	BCVA worst eye logMAR >0.1 (decimal <0.8) and best eye logMAR <0.3 (decimal >0.5)	
3	BCVA each eye/best eye logMAR ≤0.5 and ≥0.3 (decimal ≥0.3 and ≤0.5)	
4	BCVA best eye logMAR >0.5 (decimal <0.3)	
	**B. Refraction in cycloplegia**	**…/4**
1	No significant refractive error	
2	Hyperopia (≥2.0 D SE) or myopia (≥1.0 D SE) in one or both eyes	
3	Anisometropia (≥1.0 D SE)	
4	Astigmatism (>1.0 D) in one or both eyes	
	**C. Strabismus and binocular function**	**…/4**
1	Orthophoria and normal stereo acuity (TNO ≤60"; Lang =200")	
2	Heterophoria or intermittent heterotropia and normal stereo acuity (TNO ≤60"; Lang =200")	
3	No apparent deviation, or heterophoria, or intermittent heterotropia and subnormal stereo acuity (TNO >60"; Lang >200")	
4	Heterotropia and subnormal stereo acuity (TNO >60"; Lang >200") or no stereo acuity (TNO neg; Lang neg)	
	**D. Ocular structural abnormalities**	**…/4**
1	No ocular structural abnormalities	
2	Ptosis and/or epicanthic folds in one or both eyes	
3	Ocular abnormalities of the retinal vessels (i.e. increased tortuosity) and/or disc (excluding optic nerve hypoplasia) in one or both eyes	
4	Optic nerve hypoplasia (ONH) in one or both eyes	
	**Total score (A+B+C+D)** (A cut-off total score of ≥8 showed 52% sensitivity and 95% specificity)	**…/16**
